# A Nature-Inspired Hybrid Heuristic for Orchestrating the Self-Deployment of Mobile Supply Robots in Communication-Contested Environments

**DOI:** 10.3390/biomimetics11090612

**Published:** 2026-09-01

**Authors:** Fabrice Saffre, Hanno Hildmann

**Affiliations:** 1 Technical Research Centre of Finland (VTT), 02150 Espoo, Finland; 2 The Netherlands Organisation for Applied Scientific Research (TNO), 2597 AK Den Haag, The Netherlands; 3 Smart Sensor Systems Research Group, Department of Mechatronics, The Hague University of Applied Sciences (THUAS), Rotterdamseweg 137, 2628 AL Delft, The Netherlands; hanno.hildmann@tno.nl

**Keywords:** logistics, nature-inspired, self-organization, uncrewed ground vehicles, spatial distribution, contested environments, k-means

## Abstract

We present a nature-inspired approach for self-organizing logistics in contested environments characterized by uncertainty. The presented approach leverages territorial partitioning principles observed in natural collectives and systems. The logistics challenge addressed involves autonomously deploying a fleet of uncrewed mobile robots tasked with dynamically adjusting their positions to optimally service spatially and temporally fluctuating demands. Unlike traditional logistics, our approach is largely decentralized, relying almost exclusively on local interactions and decision-making. Monte Carlo simulations are conducted to evaluate performance across different scenarios, varying in client distribution (from uniform to highly clustered) and the range of the local perception of the logistic platform. Results demonstrate that the decentralized allocation strategy can deliver logistics support at a performance on par with a traditional clustering method, such as k-means, at runtime and without preliminary offline calculations.

## 1. Introduction

Logistics—the coordinated movement and management of goods, resources, and information—is a fundamental challenge faced across various sectors, ranging from commerce [1] and healthcare [2] to humanitarian aid [3] and industrial operations [4,5,6]. At its core, logistics deals with efficiently delivering the right resources, at the right time, to the right place, often under conditions of uncertainty [7], limited information, and in a rapidly evolving world [6]. As our society becomes more connected, the complexity of logistic interactions keeps growing [4]. Indeed, the field of logistics is inherently dynamic and partly unpredictable [8]. In an increasingly globalized economy, interwoven supply chains continue to grow in length [1].

A common problem in logistics is the dynamic and partly unpredictable nature of supply chains [9]. As operations unfold, points of demand can move, expand, contract, or shift unpredictably [3,5], resulting in an ever-changing distribution of the logistical support required (e.g., fuel, consumables, spare parts, or medical supplies). Handling these variations requires an adaptive support infrastructure capable of repositioning itself, ideally in real-time, to align with fluctuating needs [10].

Relatively simple disruptions or changes (such as the blocking of a major canal or hostilities along a specific coastline) can have global implications [11] for trade routes and therefore for industrial productivity [11] and, ultimately, consumer cost [12]. Traditional logistics management strategies, which rely heavily on centralized planning and static optimization models, often struggle under these conditions, leading to inefficiencies, delayed responses, resource shortages, or oversupplies.

### 1.1. Focusing on Rapid Surges in Demand

Such traditional approaches or strategies are suboptimal when it comes to rapidly supplying sudden surges in demand (for example, toilet paper despite there being warehouses full of supply in close proximity to the point of sales). Especially, when there is a very high demand that is known to occur but for which the exact time and location are unknown, it can be of great benefit to pre-emptively allocate supply to be moved into opportune positions for potential rapid delivery. This is the case for, e.g., predicted hurricane warnings where the location and time of landfall are unknown [13] or when a seasonal pandemic such as influenza is almost certain to occur [14] but for which it is very difficult to predict the actual temporal behavior sufficiently in advance to order vaccines to be delivered [14]. In both of these examples, the task of maintaining public order can be greatly facilitated by appearing prepared and being able to address the initial shortages right away. For example, being able to immediately roll out first aid supplies projects the aura of preparedness far further than these first steps and can go a great way toward ensuring public support for interventions and emergency regulations.

This, however, requires the ability to pre-emptively distribute and pre-allocate resources as well as a mechanism to maintain supply in the face of a highly dynamic environment, which may make central decision-making prohibitively computationally expensive or even infeasible in cases where individual decisions largely depend on local circumstances (which would make a policy approach unfeasible).

### 1.2. Adaptive and Dynamic Pre-Emptive Resource Allocation

Efficient logistical operations require the ability to dynamically adapt [15] and re-allocate resources [16] according to continuously changing circumstances. This responsiveness typically demands highly flexible and adaptive [8] approaches that can benefit from a decentralized approach [17], as this adds robustness against incomplete or outdated information. Centralized logistics control [18], while efficient under predictable conditions, tends to struggle with latency, scalability issues (especially with regard to growing communication overhead) [18], and rigidity [19] in rapidly evolving scenarios [6]. Decentralized logistics [18,19,20] solutions, on the other hand, promise higher responsiveness, adaptability [15,16], and resilience [5] by deferring local decision-making to individual logistical robots or vehicles [21].

However, decentralizing alone is not sufficient; the fleet of robots must be able to operate as an autonomous whole. This can be achieved through robust strategies that enable self-organization among logistics agents [22]. Bio-inspired approaches provide powerful inspiration, leveraging principles observed in natural systems to develop solutions capable of self-organizing dynamically. Nature offers abundant examples of decentralized [23,24,25] yet highly effective logistical behaviors [23,25], from the collective foraging strategies of ant colonies [26] and beehives [27] to territorial patterns of predators [28] efficiently covering vast areas without centralized coordination [28]. These biological systems naturally evolve robust solutions capable of handling environmental uncertainties [24], resource scarcity [16], and shifting demand patterns [25].

### 1.3. A Potential Solution

Bio-inspired approaches offer a promising approach to complex logistics problems [15]. Approaches like *stigmergy* [29,30]—a mechanism where agents indirectly coordinate through local environmental cues—territorial competition and partitioning inspired by animal populations, or self-organized spatial distributions found in ecological systems [23] can lead to scalable and adaptive logistics solutions [31]. Bio-inspired logistics methods not only aim at addressing the issue of dynamic demand management [15] but also the optimal utilization and positioning of limited logistical resources [16]. By decentralizing the logistical decision-making, the systems can organically respond and adapt to emerging patterns of demand [15], naturally balancing the distribution of resources [16] across areas of need without explicit central planning.

### 1.4. Motivation

Logistics in highly dynamic environments, such as defense operations, disaster relief, and humanitarian aid, poses significant challenges due to (a) rapidly changing conditions, (b) unpredictable demands, and (c) limited communication infrastructure or when signatures (actions that can be observed or detected) must be minimized (in the case of contested environments). Traditional approaches are predominantly centralized, which, while efficient in static and predictable contexts, often fail to provide the responsiveness, flexibility, and robustness needed to operate under these challenging conditions. Therefore, there is an interest in novel solutions that enable increasingly autonomous and adaptive operations by individual robotic platforms deployed to address logistical demands. Nature-inspired approaches often featuring decentralized decision-making and have been developed with promising results in the past.

### 1.5. Intended Contributions

This paper introduces and evaluates a nature-inspired approach that takes inspiration from the territorial partitioning observed in nature. Autonomous logistics robots dynamically self-organize to adapt to, and address, the spatial distribution of demand. We emphasize three contributions: First, we propose a novel decentralized algorithm enabling autonomous logistics robots to dynamically self-organize to initially unknown (spatial) demand distributions. Second, we validate the effectiveness and robustness of the proposed approach through extensive Monte Carlo simulations, demonstrating its adaptability across various scenarios where we specifically focus on the spatial distribution of the demand (uniform or clustered). Thirdly, we provide a comparative analysis between our nature-inspired solution and a traditional clustering method: k-means clustering. These three contributions provide, and validate, the presented approach.

### 1.6. Scope and Structure of This Paper

This paper applies inspiration from nature to decentralized logistics, presents a novel algorithmic approach, benchmarks simulations, and discusses results validating the proposed method. In the next section (Section 2) we first introduce the problem and then illustrate it with a few use-cases in Section 3. We then take a look at similar challenges in nature and how the solutions found there could be of use or interest to us in Section 4 and move on to drafting a solution to our problem in Section 5. We discuss our methodology and models in Section 6, and the results obtained are discussed in Section 7. We end with a summary of what was conducted and achieved in the conclusion (Section 8), as well as providing a bibliography.

## 2. Problem Statement

The operational environment for logistics in a number of use-cases (see Section 3) is inherently volatile. The fact that mobile robots of various types (aerial: Uncrewed Aerial Vehicles (UAVs); ground based: Uncrewed Ground Vehicles (UGVs), water based: Uncrewed Surface Vehicles (USVs)) are rapidly becoming (a) commercially available, (b) significantly cheaper, and (c) increasingly capable of fully autonomous operation suggest addressing logistics issues using collectives of uncrewed systems.

Simply stated, the challenge is for logistics resources to autonomously deploy (and amend their positions) to match shifting demands. The objective is to dispatch a variable number of autonomous logistics robots from an arbitrary base location into the field so that all clients there are serviced. Whether a client is or is not supplied depends on two criteria: (1) there is a logistics robot within a maximum range $R_{\max}$ and (2) this robot still has available supply, i.e., that it does not service more than $N_{\max}$ units. Note: this paper only investigates the influence of $R_{\max}$ (and certain key external parameters, such as the clients distribution); the study of $N_{\max}$ will be the subject of future work.

We consider scenarios in which an initially known area may contain a possibly large number of localized demand-generating entities (henceforth *clients*). These clients are expected to be static for all intents and purposes, and their status (whether they are currently supplied or whether they still need to be supplied) as well as position in the area are known as being situated in a central *base* or a headquarter. The base operates a fleet of uncrewed—and potentially fully autonomous—delivery robots, henceforth referred to as *servers*. Depending on the use-case, these can be, e.g., trucks with onboard UAVs to deliver supplies or UGVs [32] with wireless routers providing a service to clients within the coverage area of their Wi-Fi. Clients and servers are both capable of communicating, but this is assumed to be local communication only and to always occur between clients and a robot, meaning that robots do not exchange information between each other. Therefore, robots can only learn about other robots indirectly (when, for example, a client communicates that it is currently supplied by another robot which is currently at distance *x* from it) and are otherwise agnostic to their existence.

Upon leaving the base, robots follow some initial indication they are provided with, in this case a heading to follow, but otherwise operate independently and, for all intents and purposes, fully autonomously. In this scenario each robot *s* could be seen as transitioning through a number of phases such as ($A_{s}$) deployment (being released into the unknown environment with some initial guiding), ($B_{s}$) positioning (where the robot has started to sense relevant information about its environment, namely the existence of clients within its vicinity, and starts to inform its movement decisions accordingly), and ($C_{s}$) active (where the robot is *open for business* and acts as a server for clients in its vicinity). Note that we ignore aspects such as an upper limit for associated clients or bandwidth of the provided service, as well as limitations on the supply the server offers. Future work/extensions of our investigation could consider, e.g., phases such as ($D_{s}$) disentangling (where a robot sheds its clients by handing them over to other robots where possible), ($E_{s}$) return/re-supply (where the robot returns to a base for refueling/re-stocking/etc), and possibly ($F_{s}$) maintenance (where the robot is undergoing overhaul) before re-entering service and becoming available for (a) deployment.

### 2.1. The Base

While we generally do not want to restrict the number of bases to a one, the work presented in this article does so. We argue that the approach would work equally well with multiple bases, assuming that they could share information (and thus avoid sending multiple robots to the same demand cluster without knowing it).

There would be some potentially interesting investigations with regard to the placement of the bases or in the case of assuming that there are limited robots available in each base. However, as stated above, these are extensions to the work presented, and with clarity and brevity in mind, we restrict the number of bases to one.

In our case there is a single base, which has a fleet of robots (which in our example are UGVs, but could also be UAVs). The base (a) knows of all clients in the field (including their locations) and has a (not further explained) insight into (b) the overall demand in the area and (c) the currently unmet demand (including the clients that are experiencing this shortage). While this might constitute a leap of faith with regard to the realism of the formal model, we argue that this is an appropriate formalization given that we aim to keep the investigations and the approach broad and thus broadly applicable. There are many means and constructions to enable some understanding of the situation for the base. This is discussed a bit more in detail for the specific use-cases (cf. Section 3) as well as for their inspiration from nature (cf. Section 4.1) and are not further specified at this point.

### 2.2. The Server Robots (Supply)

**Figure 1:**
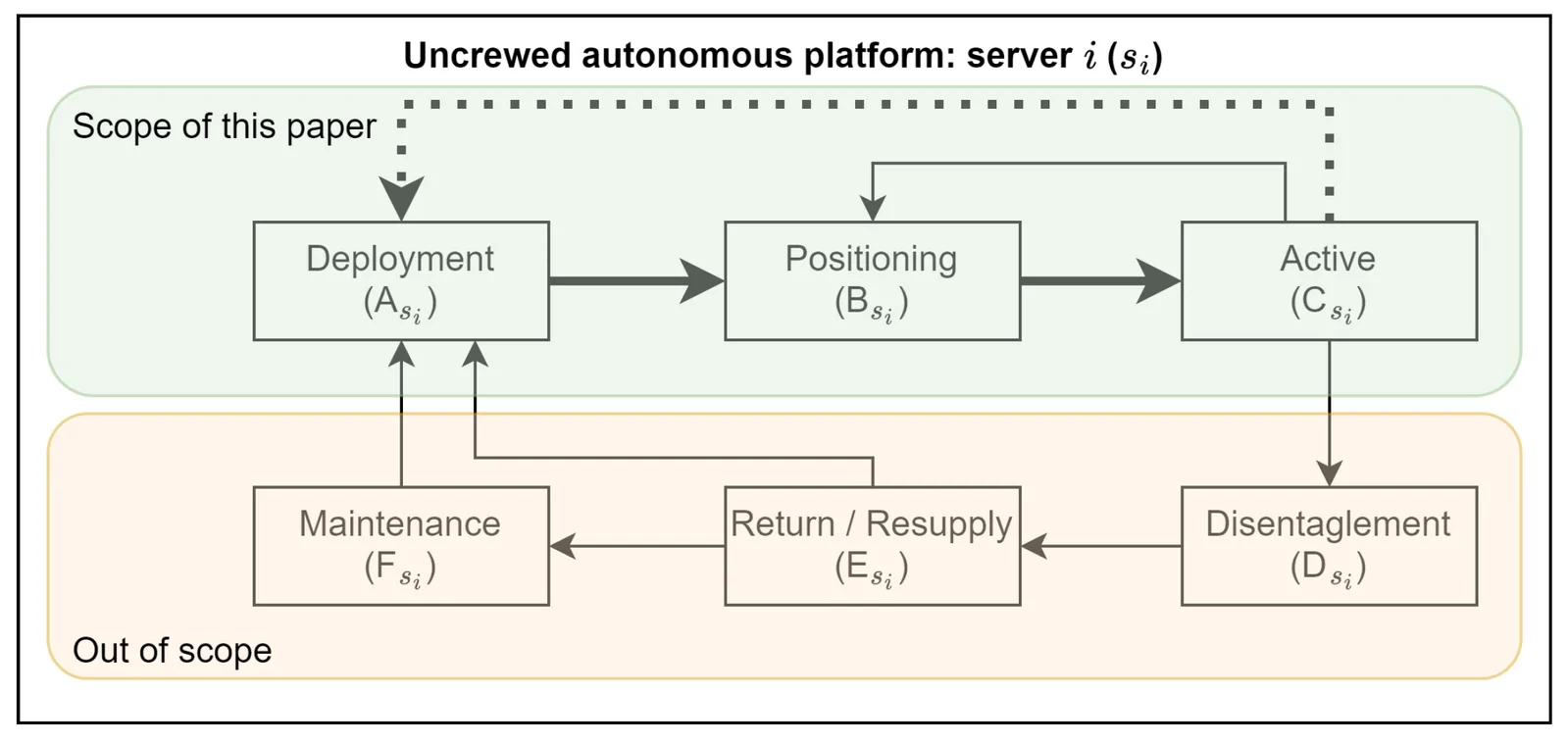
The work discussed in this article only considers the allocation and re-allocation problem of servers. This covers the deployment of a robot, its movement through the environment, and its eventual operation as a supply generating/providing/offering entity in the environment; see Figure 4 for details. The practical issues of removing the robot properly from ongoing operations and its return to the base for re-stocking or maintenance are not treated in this article.

The servers are considered to be mobile robots capable of autonomous operation. This means that the robots can move under their own power and have the ability to make decisions without any supervision or intervention. This means that once deployed and provided with initial instructions, the robots can then fully autonomously move through the environment until they themselves decide to change from the positioning phase to the active phase (see Figure 1) in which they start operating as a server.

Robots are expected to be able to sense their environment and to communicate with clients. The former refers to the ability to perceive clients, while the latter means that the robot is able to exchange rudimentary information with any client that it can perceive; both of these capabilities are subject to range $R_{\max}$ (which for simplicity is circular in our model).

Therefore, the overall problem of supplying all clients in an area is solved in a fully decentralized manner by the autonomous platforms in an area. This also means that there is no communication other than local communication, which is subject to a maximum range.

### 2.3. The Clients (Demand)

Clients are demand-generating locations in the area. Clients are fixed (immobile), and for the sake of simplicity the demand is not further quantified. Once they are within the reach/service range of a server, the clients are assumed to have their demand satisfied.

In the case where multiple servers are available (within range of the client), the client will autonomously allocate itself to the closest server. Clients are assumed to communicate basic information to all servers within range at regular intervals, specifically communicating (a) their location and (b) their status (which is either supplied or unsupplied).

## 3. Potential Use-Cases

The decentralized and self-organizing logistics system presented here offers significant potential across various dynamic real-world scenarios. Below, we explore four variations of our problem (cf. Section 2) as use-cases to highlight the versatility of the problem as well as the flexibility and effectiveness of the proposed approach. Note that two of the four use-cases discussed below (namely those considering either continuous operations or the defense oriented scenario, as discussed in Section 3.3 and Section 3.4), are only partially covered by the implemented model as they extend to including re-supply, which we explicitly exclude from the work reported on in this manuscript.

### 3.1. Providing a Service Such as Wireless Access in the Absence of Fixed Infrastructure

Let us start by considering a scenario where the to-be-supplied resource is a service, such as wireless internet connection through access points. In this example there is no physical object that has to be delivered and handed over; instead, there is a service (limited by a range) that is provided to clients within the coverage zone. Note that we do ignore the signal attenuation and the issues such as the fact that a client further away from the server will incur a higher cost for the communication with the server due to the higher energy requirements to broadcast a signal over the longer distance.

In this scenario, robots are dispatched into the general directions of expected demand clusters. Once released, the robots first move into position and then continuously amend their position so as to, collectively, service all clients. While this description covers moving clients as well, we keep it simple and think only of static clients such as, e.g., brick and mortar businesses, operation centers, and staging areas for relief operations. The contexts of this scenario are the aftermath of a disaster where infrastructure is wiped out or in preparation for a large scale event such as New Year’s Eve.

The advantages of such a system are evident: as the robots never stop trying to improve their positions, the fleet continuously optimizes its operation. In addition, the fleet can react to the movement of clients or changes in demand (new clients appearing; old clients disappearing). This adaptability significantly enhances the efficiency, responsiveness, and resilience of the offered service across an entire area, which is crucially important in disaster relief scenarios, refugee camps, and large-scale public events.

### 3.2. Medical Supply Distribution in Anticipation of Disasters

Secondly, we consider the allocation of physical resources needed for rare specific events, such as emergency response materials needed in the aftermath of a natural disaster (e.g., hurricanes or floods) or anticipated large-scale event (e.g., New Year’s Eve). In such scenarios, medical resources, first aid supplies, blood transfusions, and emergency equipment must be distributed proactively to optimize response times and save lives. Unlike traditional logistics operations that match supplies to predetermined client lists, this scenario requires flexible and responsive provisioning of general-purpose resources based on expected but uncertain demands. With the supplies pre-emptively distributed across the operational theater, the second stage of such an operation is then the continuous updating/restocking/re-supplying of the resource distribution as the event unfolds and demand for the resource is created.

This fits our problem description in that we have a base with robots that can be deployed in advance into an environment with expected demands. By positioning (and re-positioning) the supply robots in a manner that aims at equalizing the access times for all served clients, a somewhat fair as well as timely distribution of the supply can be warranted for the period when a sudden jump in demand otherwise is expected to overwhelm clients in unpredictable ways.

Assuming that the actual need of a specific client is hard to predict, then the ability to pre-emptively ensure equal means to access supplies at roughly the same cost (time and physical distance) enables planners to hedge against massive jumps in demand.

### 3.3. Continuous Operation with Cyclical Supply

The third use-case involves continuous operational environments, such as construction sites, remote industrial operations, or persistent humanitarian aid missions. In such scenarios, resources are consumed continuously, and supply replenishment must occur cyclically. The dispatched autonomous logistics robots must perform as in the examples above but once depleted will have to return to a base to restock. This means that the resulting use-case, while similar to the previous example of pre-emptive allocation of resources, adds the matter of switching out servers and having robots return to base to restock (though this part of the autonomous operation is not included in our investigations yet; cf. Figure 1). Practically, this means that the scenario extends the second use-case by dispatching robots multiple times. This allows for the scenario to be dynamic in the distribution of the clients as well. For example, one region of the area which requires many servers in the beginning may later be built-up and require only supplies for maintenance (as opposed to construction), while another region now experiences peak demand. Moreover, this approach inherently handles variations in consumption rates across different regions, dynamically shifting the concentration of supply robots to match evolving local demand.

This scenario clearly still sits inside the problem description (cf. Section 2) provided, but it requires the base to continuously re-stock robots and to re-deploy them, albeit to potentially different locations. This means that the autonomous operation of the robots themselves can be seen as repeated but entirely separate deployments, because no memory of previous missions is kept by a robot.

### 3.4. UGV-Supported Frontline Logistics for Combat Operations

As a last use-case variation, we want to discuss the soon-to-be reality of uncrewed systems and autonomous robots operating as collectives (or swarms) in the domain of defense. Clearly there is a potential for the deployment of uncrewed systems in environments that are heavily contested and where the deployment of human personnel would come with a sizable danger to life or limb. But while it is evident that there is potential gain to be had in considering uncrewed systems, there is also a lot of trouble to be found, not the least in the realms of ethics and from international legal frameworks and agreements. Therefore, this article and the work discussed steers well clear of offensive combat operations; we do not discuss uncrewed or autonomous weapon systems here. The ethical use of autonomous platforms is at this point a broad and widely discussed topic. While the authors have some expertise in this area, we feel that the discussion thereof within this manuscript is outside of the scope. The argument made is as follows: (a) we discuss the operation of logistics platforms without any offensive capabilities, so the use of autonomous weapon systems is out of scope, and the ethical use of self-driving systems is widely discussed in the literature and does not play into the work we present; (b) the work presented here focuses exclusively on the distribution and the positioning of the platforms and intentionally ignores details of how these platforms actually move in the real world.

We also do not discuss whether the platforms are operating in well-regulated road networks, under which legal system (Europe or the US, e.g.) or operational constraints (day-to-day or during a natural disaster) they operate, or whether the platforms are wheeled, tracked, or legged. We assume the use of unarmed UGVs, such as some of the systems offered by Milrem [33], which are large enough to carry position, navigation and timing (PNT)-solutions that do not rely on GPS. Such alternatives can be other global navigation satellite systems (GNSS)-based approaches such as, e.g., using Low Earth Orbit (LEO) mega-constellations such as Starlink [34]. Such robots can navigate entirely autonomously and can carry significant amounts of cargo (e.g., backpacks weighing 40 kg for infantry soldiers).

Note that medical evacuation through unmanned robots is an extension to the problem that is intentionally not considered here. While considering the pre-emptive stationing of medical evacuation robots in a combat zone is interesting it would be rather naive to simplify the MedEvac challenge to match the problem description given in Section 2. We therefore specifically restrict our use-case scenario to the transport of resources (e.g., backpacks, ammo crates, and heavy ordnance) and the desire to place the robots in opportune locations. Note that we are ignoring the challenges associated with having a re-supply location that may be especially attractive for opponent artillery shelling; it is straightforward to see how such mobile cargo mules can offer great benefits when it comes to enabling the soldiers to move faster and stealthier and generally for shortening the distance a soldier has to travel in order to reach a re-supply point.

It should also be noted that in the military domain (a main area of focus given the conflict in Ukraine), logistics works along fundamentally different priorities: while civilian logistics is generally focused on minimizing resource consumption and optimizing operational efficiency, the military has to operate in a contested and adversarial environment. Due to this, platform loss and adversely changing environments are major challenges, and the primary performance measure is often the delivery of the required freight to the right location and at the right time. Often, there is a significant tolerance for suboptimal resource consumption as long as these performance targets are met.

### 3.5. Overview and Comparison of the Use-Cases

We briefly compare the four discussed use-cases in Table 1. Among the most relevant differences in these scenarios is the service or capability provided by the logistics robots: in the case of offering, e.g., a wireless access point (as discussed in Section 3.1), the robot can position itself such that the average distance between it and the users is minimized (since the cost of connecting to a wireless router is a function of the distance between client and server). On the other hand, if the robot provides logistics support in the original sense, then a physical product needs to be transported and exchanged with a client. This means that the physical accessibility to the robot is a relevant factor.

In addition, the product or service provided by the robot itself poses operational limitations: while a wireless access point delivers a product that does not have physical dimensions or is carried by the robot itself, this is different for a robot delivering packages, since in order to re-stock the robot has to physically return to a warehouse.

The use-cases in Section 3.1 and Section 3.2 offer examples for a system providing either a stock of products or a service. The remaining two use-cases could deliver either or both. The difference between the first two and the last two use-cases is how we evaluate their overall performance: while providing wireless access or delivering products, the performance of a robot and the entire fleet is evaluated within a single deployment, while the last two use-cases require operation over time and to also cover the deployment of additional robots to replace the depleted ones in the field.

### 3.6. Where This Problem Exists in Nature

This challenge of dynamic resource allocation and territory partitioning is not unique to human-designed systems but has long existed in nature [23,28]. Biological organisms routinely manage complex logistical tasks [16,25] with remarkable efficiency [35] and robustness [24,25], despite relying exclusively on local information [23,36] and decentralized decision-making [23,24]. The study of bio-inspired logistics therefore offers potential strategies for managing resources in uncertain and dynamic environments without relying on centralized planning or the exhaustive availability of global information.

We argue that a nature-inspired, decentralized logistics framework that can operate without dependence on persistent and reliable communication links has the potential to offer robust, scalable, and highly adaptive approaches for complex and dynamic logistical challenges. The versatility of the approach, demonstrated through the results discussed in Section 7, suggests that even after tailoring the generic solution (proposed in Section 5) to a specific use-case, it will retain its robust and adaptive nature.

## 4. Bio-Inspired Logistics

The goal of the logistics robot swarm is to optimally supply all clients, i.e., to ensure that enough supplies are available at strategic locations while avoiding inefficiencies such as multiple servers converging unnecessarily to the same area and causing over-provisioning. A uniform distribution of resources, or a dynamic regular mesh of supply points, typically falls short of meeting operational demands effectively.

There is a well-documented natural phenomenon that provides a decentralized solution to a very similar problem: the partition of space into multiple mutually exclusive territories by competing organisms [28]. When resources [37] are approximately homogeneously distributed and all competitors have similar strength, territories tend to have the same size and form a semi-regular pattern [28] (e.g., a Voronoi [38] tessellation [28]). However, in the absence thereof (resource distribution is uneven and/or the organisms consuming them have variable needs or strength), boundaries fluctuate (some territories expand, while others shrink and/or become more densely clustered).

Many animal species establish territories [39] that are dynamically adapted [37] based on local resource availability [37] and the strength or capability of the individuals or groups involved. Predators, such as wolves or lions [37], partition hunting grounds into territories to optimize access to prey, ensuring efficient resource utilization while minimizing conflicts. In these scenarios, the spatial distribution of prey and the relative strength or energy state of predators dictate territory boundaries [28].

Another natural example, though slightly more abstract, can be drawn from chemical and physical self-organization processes such as reaction–diffusion systems (e.g., the Belousov–Zhabotinsky reaction [40]). These chemical systems spontaneously form stable patterns [41] based on entirely decentralized local interactions [42], achieving spatial structures that dynamically adapt to local chemical concentrations and reaction–diffusion rates [43]. While lacking any intent in the decision-making process (after all, molecules have no free will), the fundamental principle of decentralized local interactions results in emergent adaptive spatial partitioning [23], akin to the territorial partitioning desired in our autonomous logistics scenario.

These natural examples motivate bio-inspired logistics systems for robots. Each mobile robot in the swarm can be viewed as a territorial competitor, where the supplies it offers (e.g., medical aid, food, or wireless access) are needed by clients (which represent the “territory” in this analogy). Contrary to many natural systems, our robots are not competing with each other for the size of the territory (i.e., who serves more clients) but rather cooperating to cover the entire territory (i.e., collectively service all clients) as well as to optimize each individual client’s distance to a supply source. This simplifies the mechanisms driving the self-organization, as we do not need to consider competition among robots, only the available clients. Like organisms in nature, the uncrewed robots dynamically adjust their deployment locations based on the clients they are individually aware of.

Motivation for adopting bio-inspired principles arises from nature’s proven efficiency in solving complex logistical problems [15] without centralized control [23,24,25], extensive communication overhead [44], or requiring exhaustive global knowledge [15]. Biological systems inherently demonstrate robustness [25], scalability [23], and flexibility [16,24], efficiently managing resources despite uncertainty [29], environmental variability [24], and limited communication [23,29]. By emulating these natural behaviors, engineered autonomous logistics systems can achieve higher adaptability, responsiveness, and robustness, overcoming the limitations inherent to traditional, centrally managed logistics systems [18].

### 4.1. Use-Case-Specific Analogies and Inspiration

Despite the differences between biological ecosystems and human-engineered systems, several fundamental similarities make bio-inspired strategies especially valuable for logistics. Both involve managing scarce resources, coping with uncertainty and incomplete information, adapting dynamically to changing environmental conditions, and requiring robustness to disturbances. By leveraging principles such as local self-organization [23,29], positive feedback [25], and adaptive task allocation [16,23,45], bio-inspired logistics methods can enhance the performance and resilience of decentralized logistic systems [31].

#### 4.1.1. Rolling out a Pervasive Consumable Service

With regard to providing consumable resources to an area (see Section 3.1 for this use-case), we envision bio-inspired self-organizing logistics, where autonomous robots (e.g., mobile power generators, communication relays, or water purification units) independently and continuously adapt their deployment positions and can do so in the absence of reliable communication between the robots. They assess local demand intensity through local communication with the consumers. Based on their own resource capacities, robots dynamically form service “territories” that reflect immediate localized consumption needs. This self-organized territorial partitioning ensures that robots position themselves optimally based on real-time information available to them locally rather than relying on outdated predictions or centralized and high-level orders.

#### 4.1.2. Pre-Emptive Supply Distribution in Anticipation of Disasters

For the use-case of pre-emptively distributing medical supplies, discussed in Section 3.2, we consider the scenario of an impending hurricane. In such situations, medical facilities and emergency responders know approximately where the demand for medical resources will surge, but precise locations and quantities remain uncertain. Furthermore, the transportation infrastructure may be adversely affected by the hurricane, but it is impossible to predict how or where. A decentralized logistics deployment inspired by territorial competition found in nature allows autonomous logistical robots to pre-position themselves strategically. Each robot dynamically selects its deployment location based on anticipated demands, local population densities, or projected risk areas, continuously adjusting its position as updated information becomes available.

Consequently, when the hurricane strikes or mass casualty incidents occur, supplies are already distributed in a manner that optimally enables the adjustment to the situation on the ground, minimizing logistical delays, ensuring a rapid response capability, and substantially improving the overall resilience and effectiveness of disaster response.

One advantage of this decentralized [18] and adaptive [8] approach is that it inherently mitigates risks associated with incomplete information and uncertainty. Robots do not rely on comprehensive or precise predictions from centralized authorities, which might be delayed or simply inaccurate. Instead, each autonomous logistical unit continually assesses and reassesses local conditions based on immediate surroundings and the updated status of client needs, collectively and organically forming a robust and resilient supply network.

#### 4.1.3. Pervasive and Persistent Logistics During Ongoing Aid Operations

Consider an ongoing humanitarian relief operation in a remote area (the use-case discussed in Section 3.3): medical teams, food distribution points, and shelters continuously require replenishment of supplies. The challenge is to maintain uninterrupted service efficiently, minimizing downtime and travel distance. Bio-inspired logistics algorithms provide an elegant solution, enabling each autonomous logistical robot to dynamically manage its service area, balancing its local resource provision against ongoing consumption rates. As individual supply robots exhaust their stocks, they autonomously return to the central depot for replenishment, while newly stocked platforms simultaneously deploy into the operational field. This ensures uninterrupted supply coverage and effective management of resource distribution cycles.

#### 4.1.4. Combat Logistics

Combat in ant colonies typically involves territorial defense, offense, or inter-colony competition over resources (e.g., food or territory). During combat, ants must efficiently coordinate not only combatants but also logistics: reinforcing the frontline, redistributing troops, evacuating wounded, and provisioning combatants with food.

Leafcutter ants [46], for example, face frequent territorial disputes and invasions by competitors [47], predators, and parasitic species. When this happens, efficient logistics are essential for the survival of the colony. Combat logistics in these ants typically involve one or more of the following: transporting combatants (reinforcements) and materials [47] (e.g., debris, soil for fortifications, or defensive barriers) rapidly to front lines, evacuating injured ants [48] back to safe zones or the nest, and generally provisioning frontline ants (primarily with liquids, nutrients, or fungally derived food) [46] to maintain sustained defense. All of these are strongly situation-dependent, and the ants in question manage to optimize their activities so as to deliver near-optimal logistics. This makes the approaches used relevant for the defense use-case introduced in Section 3.4.

A common problem in defense logistics is the dynamic and unpredictable nature of supply chains [49]. As operations unfold, the frontline moves and combat units advance, retreat, or regroup, whereby they change the demand for logistical support (ammunition, fuel, field rations, medical supplies, etc.). Accommodating this requires an adaptive support infrastructure, capable of redeploying itself in real-time to follow operational needs, e.g., by moving a mobile depot forward or backward in response to changing circumstances.

Robots could be used to keep frontline units supplied, thus freeing human personnel for more demanding tasks. One concept involves deploying self-organizing UGVs [32] as mobile stores, capable of following troops while carrying equipment and supplies. These mobile robots would effectively be acting as the burden animals of old (“mules”), only with much enhanced information processing capabilities and the ability to act autonomously.

Taking this concept to its full potential involves a robotic logistics force managing its own resources (individual robots at variable store capacity) such that combat unit support is optimized by ensuring that the right supplies are available in sufficient amounts at easily accessible locations (e.g., close to the troops that need them). It is paramount to avoid costly mistakes such as dispatching multiple supply robots to the same region of the frontline, thus over-provisioning some combat units while leaving others unsupported. However, it is equally critical to ensure that when the supply needs are highly concentrated (e.g., because there is a localized breakthrough or enemy offensive) this is reflected in the deployment of field logistics. This entails that an even distribution of resources in space will often be suboptimal and that establishing a regular mesh of mobile depots, even a dynamic one, will rarely meet operational requirements.

### 4.2. Summary

There are many examples where nature exhibits decentralized logistic strategies. By studying the underlying models we can address similar challenges in artificial systems. The territorial partitioning approach is one such promising bio-inspired method that addresses the critical problem of dynamic resource allocation. It seems to fit all of the use-case variations we discussed as instances of the generic problem description we provided. By adopting decentralized decision-making strategies inspired by territorial organisms, logistics systems can become more responsive, robust, and efficient, significantly improving their ability to manage resources effectively in rapidly changing and uncertain environments.

## 5. Drafting a Solution

The envisioned system proposes an innovative approach to autonomous logistics, which leverages decentralized decision-making and local communication to ensure effective and robust supply management. The restriction of long-range communication also serves as a signature reducing measure that reduces the visibility of the robots. In this envisioned system, a central depot or base maintains an overarching awareness of the client distribution—specifically their fixed locations—and the current supply status of each client. Crucially, the base does not maintain direct control or communication with the autonomous supply robots after their initial dispatch. Instead, once autonomous mobile supply robots are deployed into the operational environment with a tentative direction, these robots become entirely self-managing and autonomous, relying solely on local interactions and direct client communications to organize and optimize their supply territories.

The autonomous robots themselves operate independently, endowed with the capability to sense and communicate within their local surroundings. Upon deployment, each robot initially follows an impulse provided by the depot based on the immediate known distribution of unsupplied clients. Once in the field, however, the robots rely solely on local sensing to continually reassess and re-position themselves.

**Figure 2:**
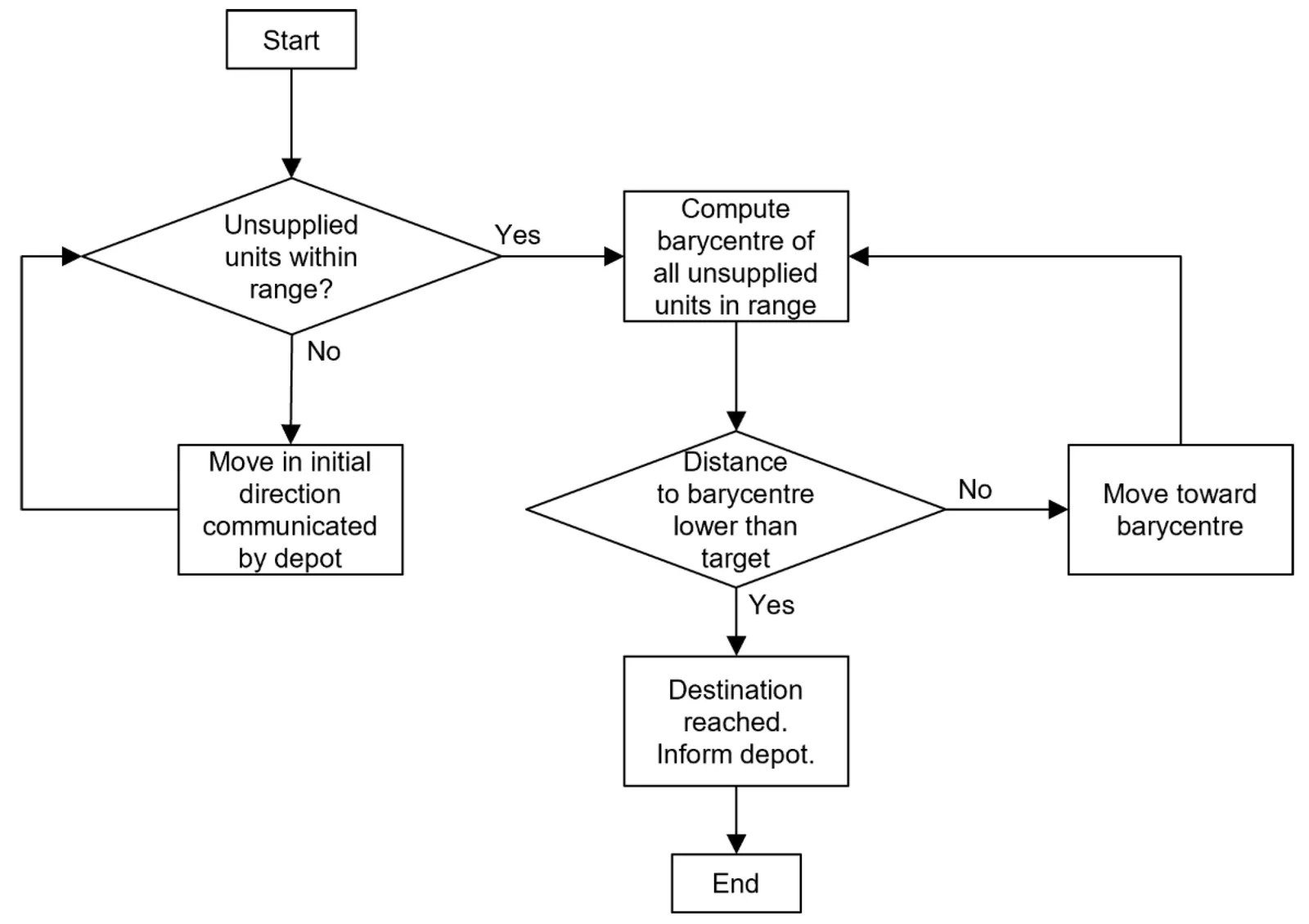
The operation of each individual robot, shown as a flow chart.

Robots become aware of nearby clients, detecting their locations, current status (supplied or unsupplied), and the distance to each client within a certain detection range. They dynamically adjust positions based on local information (cf. Figure 2), seeking an optimal spatial arrangement in response to ongoing changes in supply demands.

Clients, in turn, remain stationary and communicate their current supply status and their relative distances to nearby robots. Notably, the decision regarding which logistics robot services a client is made by the clients themselves—although, practically, this decision is deterministically driven by proximity, ensuring each client connects to its nearest logistics resource. Thus, the resulting emergent supply pattern is entirely decentralized, self-organized, dynamically adaptive, and signature aware (remember that we specifically mentioned the ability to reduce the computational overhead, as well as the resulting signature, incurred from excessive communication). Such a strategy significantly reduces centralized decision-making burdens, limits communication overhead, and enhances system resilience and adaptability to changing environmental and operational conditions [18].

### 5.1. Underlying Considerations

The individual robots are governed entirely by algorithmic decisions, but the emergence of a global solution from their independent execution of the ruleset until the local end condition is met (reaching the barycentre of their respective territory) is a heuristic; and a simple hybrid heuristic was used to orchestrate the self-deployment of the logistics robot fleet. It is hybrid in the sense that it is mostly decentralized but can include an initial, centrally provided direction of movement when leaving the depot in the case that no field units are within the catchment area within the $R_{\max}$ radius (assumed in this case to be the robot’s sensing range, i.e., where it can detect, or receive indication of, the presence of an unsupplied unit). So, in short, when there are no such unsupplied units within the catchment area immediately surrounding the depot, the next logistics robot is sent toward the nearest unit outside of the corresponding perimeter, which presupposes that this information is available centrally (but not in the field).

Upon deployment the robot is entirely autonomous and makes decisions based exclusively on locally available information. The basic rule is simple: move toward the barycenter of the known unsupplied demand within $R_{\max}$ until you find yourself within a target distance (threshold) of the corresponding coordinates. Note that bots cover 1% of the edge of the square area per time-step; the threshold is the corresponding distance (i.e., 0.01). This means that a bot stops when it is within a distance of the barycenter of its territory which it could cover in under one time-step. Obviously, as it moves in the intended direction (reminder: in the absence of a barycenter because no unsupplied units are within the catchment area, it is replaced by the coordinates of the nearest unsupplied unit at the time of departure from the depot), the robot’s neighborhood (and so the collection of unsupplied units within detection range) changes, resulting in successive corrections. As a result each robot tends to move to a location where its usefulness to the collective is maximized (i.e., where it can make the biggest difference in terms of newly supplied field units). When the final location is reached (until possible future competition changes local conditions), the supply robot becomes active, and all the units affiliated to it (within radius $R_{\max}$ and closer to it than to any other previously deployed robot, see next paragraph) are supplied.

Finally, we address the competition between robots that have overlapping catchment areas. Again, a simple rule is used: once the latest supply unit to be deployed has reached its tentatively final set of coordinates, it “captures” all field units that were previously serviced by another logistics unit if and only if they happen to be closer to it than to its peer. In this event, the previously deployed unit is informed of the change (by the client), which may result in the barycenter of all the remaining affiliated field units drifting (as some have been “captured” by the newcomer). If this is the case (and the distance to the new barycenter exceeds the threshold), the corresponding robot briefly reverts to the positioning phase to relocate (see Figure 1). This can have cascading effects and, when all movement by the robots ends, a new near-optimal state is reached. The depot is then notified of this event, and a new unit is deployed if necessary, i.e., if there are still unsupplied field units. When this is no longer the case and the final state is reached (all field units are supplied and all the logistics robots are at the barycenter of their “territory”), the simulation ends, and performance metrics are collected.

### 5.2. Comparison with Traditional and Alternative Logistic Approaches

In contrast to the decentralized method proposed here, traditional logistics approaches typically involve centralized route planning [50], scheduling [51], and inventory management systems [52]. These systems assume reliable, timely communication with a central controller capable of continuously monitoring client needs and robot statuses. Centralized methods often use global optimization algorithms (e.g., Mixed Integer Linear Programming [53], Vehicle Routing Problems [54], and inventory optimization techniques [55]) to manage resource allocation comprehensively. While effective under predictable and stable conditions, such centralized methods frequently face performance issues in volatile scenarios. Any disruption or delay in communication can result in suboptimal decision-making, delayed responses, and inefficient resource utilization.

Other modern logistic solutions adopt hybrid or semi-decentralized approaches, wherein local autonomy is granted to logistics agents, but some global oversight or occasional global coordination occurs. For instance, multi-agent systems or swarm robotics algorithms, such as Ant Colony Optimization (ACO) [36], Particle Swarm Optimization (PSO) [56], or swarm-based task allocation algorithms, allow local agents considerable decision-making autonomy while still periodically synchronizing through global communication or updates. These methods balance responsiveness and autonomy with some centralized oversight, facilitating global optimization of resource allocation while maintaining robustness against communication disruptions.

The envisioned approach discussed in this work, however, emphasizes full decentralization during operation, maintaining global awareness only during initial deployment or replenishment cycles. Such an approach is particularly suitable when communication infrastructure may be compromised or communication costs prohibit frequent global updates. The use of locally determined territorial partitioning naturally mitigates the risks associated with disrupted global communication, as autonomous robots can independently continue operations based on purely local interactions.

Despite their advantages, semi-decentralized logistic systems can encounter specific challenges. Without global coordination, decentralized systems might initially display suboptimal global patterns or inefficiencies such as overlapping service areas or uneven resource allocation. Yet, nature provides evidence that such inefficiencies diminish over time as the decentralized interactions and local decisions collectively drive the system toward equilibrium. This natural property underpins the motivation for adopting nature-inspired decentralized solutions and suggests that, assuming that sufficient local autonomy is granted and clear interaction rules are provided, complex logistical networks can achieve optimal or near-optimal solutions without central control.

Therefore, logistic methods range along a spectrum from fully centralized over semi-decentralized to fully decentralized, each offering distinct advantages and disadvantages. While centralized logistics excel under stable conditions with reliable communication, their effectiveness rapidly declines under uncertainty or disruption. Bio-inspired decentralized methods, as proposed here, prioritize local interactions, self-organization, and adaptive behaviors, making them particularly resilient and effective in volatile, unpredictable, or disrupted environments. Future developments might beneficially explore integrating partial global coordination for specific scenarios, potentially blending the advantages of both centralized and decentralized logistic paradigms.

### 5.3. The Approach

The actions of the base consist of occasionally deploying a robot into the environment. When this occurs, the robot is given a heading to follow (we assume for simplicity reasons that the environment is 2D, flat, and obstacle free) but is otherwise given full authority to make its own decisions from then on out. The actions of a robot are then to initially travel in the provided heading until a client in need of supply is encountered (this initial phase is called deployment). Once that happens the server changes from the deployment phase to the positioning phase.

#### 5.3.1. The Base

The operation of the base is continuous. The base monitors the overall supply situation in the area (on the basis of the unmet demand only; over-provisioning is not considered) and occasionally deploys a server. Upon deployment, a server robot is only provided with a direction in which to move; once deployed (and moving away from the base) there is no further communication from the robot with the base itself. The assumption is that the base serves as a server-deployment station and is further unrelated to the creation and delivery of the actual demand.

**Figure 3:**
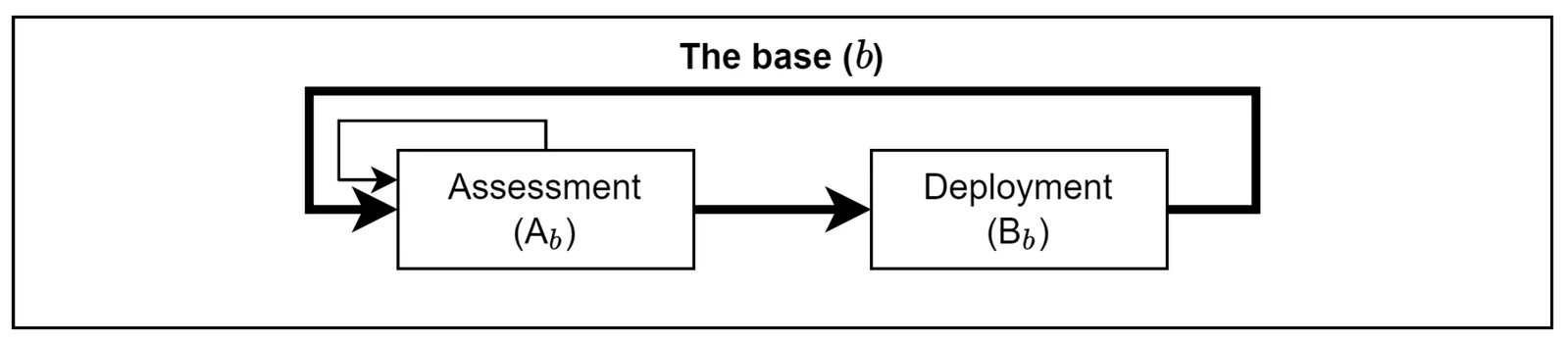
The depot or base simply monitors the overall supply situation in the area and occasionally deploys another server robot into the general direction of a shortage.

A base *b* therefore only really has two stages: ($A_{b}$) assessment (where some time may pass and where the current overall demand is evaluated); ($B_{b}$) deployment (where another robot is deployed due to under-provisioning in some area); see Figure 3.

#### 5.3.2. The Server Robots (Supply)

Robots can use the location of perceived clients (which the clients communicate to it) as well as their status (which is either supplied or unsupplied) to identify the current unmet demand within their range. Using this information, the robots can calculate a barycenter and move there. As movement may reveal new clients, the barycenter may dynamically change as the robot moves toward it. This process is, however, finite, and the robot will eventually reach a stable barycenter.

**Figure 4:**
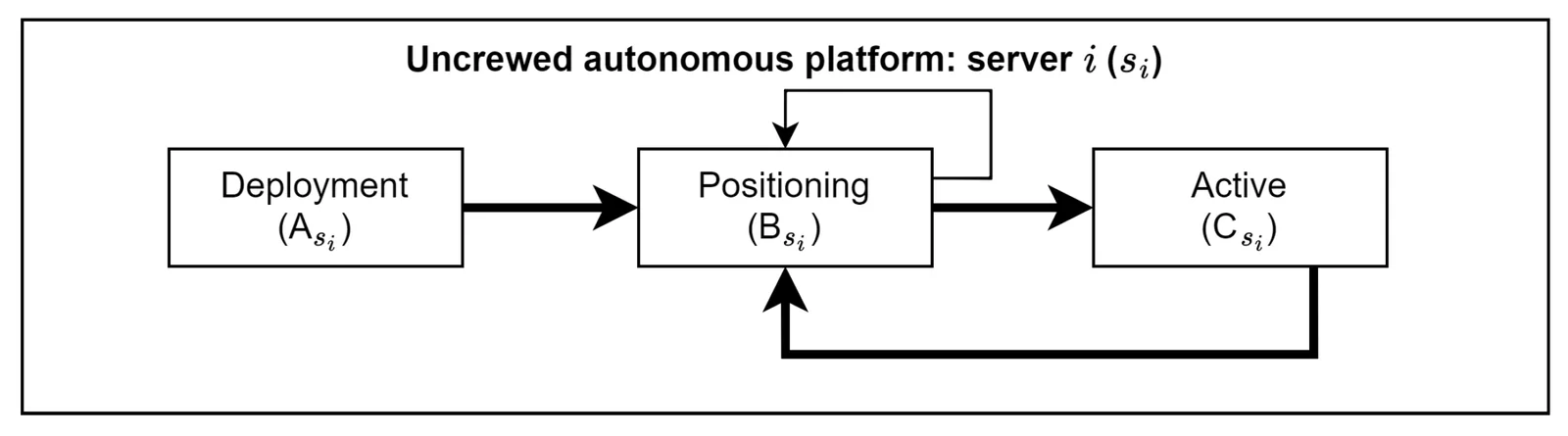
The supply UGV initially moves as instructed during deployment and eventually settles into alternating between supplying clients and re-positioning itself to reach more clients. When the supply does not move or change, this process eventually converges toward a steady state.

Once this is achieved the robot changes phases and starts its service/announces that it is open for supplying near clients. If previously supplied clients change their status (because their supply robot orphans them by departing from the area for, e.g., re-supply or maintenance) this might affect the barycenter, and when this happen, the robot may re-position itself (this in turn may orphan some clients which then may trigger other robots to move or the base to deploy another server). Figure 4 illustrates this.

**Figure 5:**
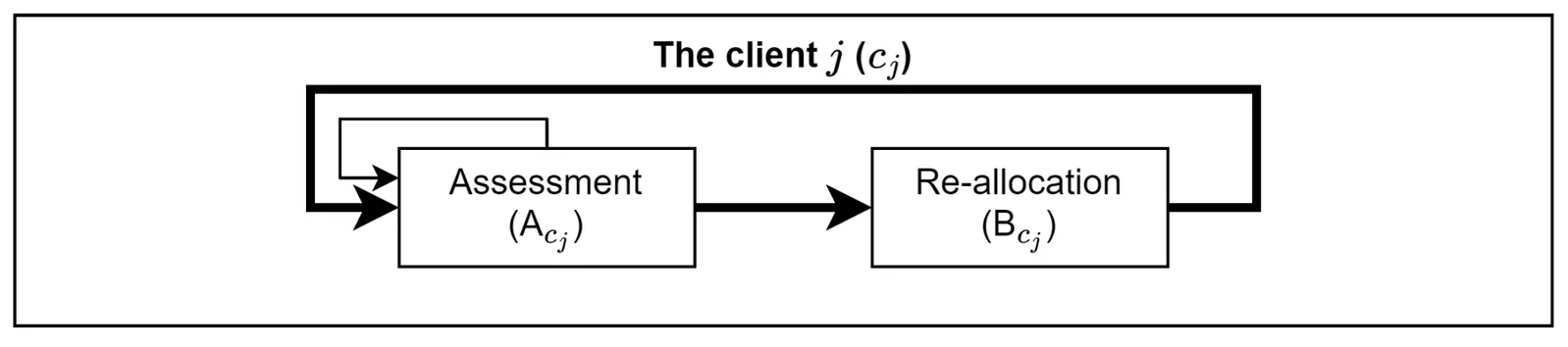
Clients occasionally re-allocate themselves if a closer supply UGV is available (thereby potentially changing the UGV’s barycenter). This process eventually converges.

It should be noted that clients (cf. Figure 5) will prefer the closest server (Euclidean distance in the real world), which means that clients currently using one server may over time shift to a recently appeared, closer one. When this happens this may affect the barycenter of the original server which may also trigger re-positioning of that robot.

## 6. Materials and Method

### 6.1. Scenario

The test scenario involves a large number of units to be supplied (N=1000), distributed in a square area (1 ${\mathrm{km}}^{2}$). This distribution can be homogeneous or aggregative. To generate the desired pattern, a selected number $N_{c}$ of cluster heads (10, 100, or 1000) are placed at semi-random locations in the environment (with the constraint that cluster heads are at least 10 m apart). Then, the remaining units (990, 900, or 0, respectively) are placed in such a way that each new one is no less than 10 m but no more than 30 m from at least one already placed peer. For 1000 clusters, this method generates a homogeneous distribution, whereas for 10, it returns 10 groups of variable sizes which may or may not overlap.

### 6.2. Objective

The objective is to dispatch a variable number of autonomous logistics robots from an arbitrary headquarters location (by default, the center of the 1 ${\mathrm{km}}^{2}$ area) into the field so that all demand-generating clients are within the supply range of a robot.

Whether a unit is or is not supplied depends on two criteria: (1) there is a logistics robot within a maximum range $R_{\max}$ that (2) does not service more than $N_{max}$ units.

### 6.3. Benchmark

**Figure 6:**
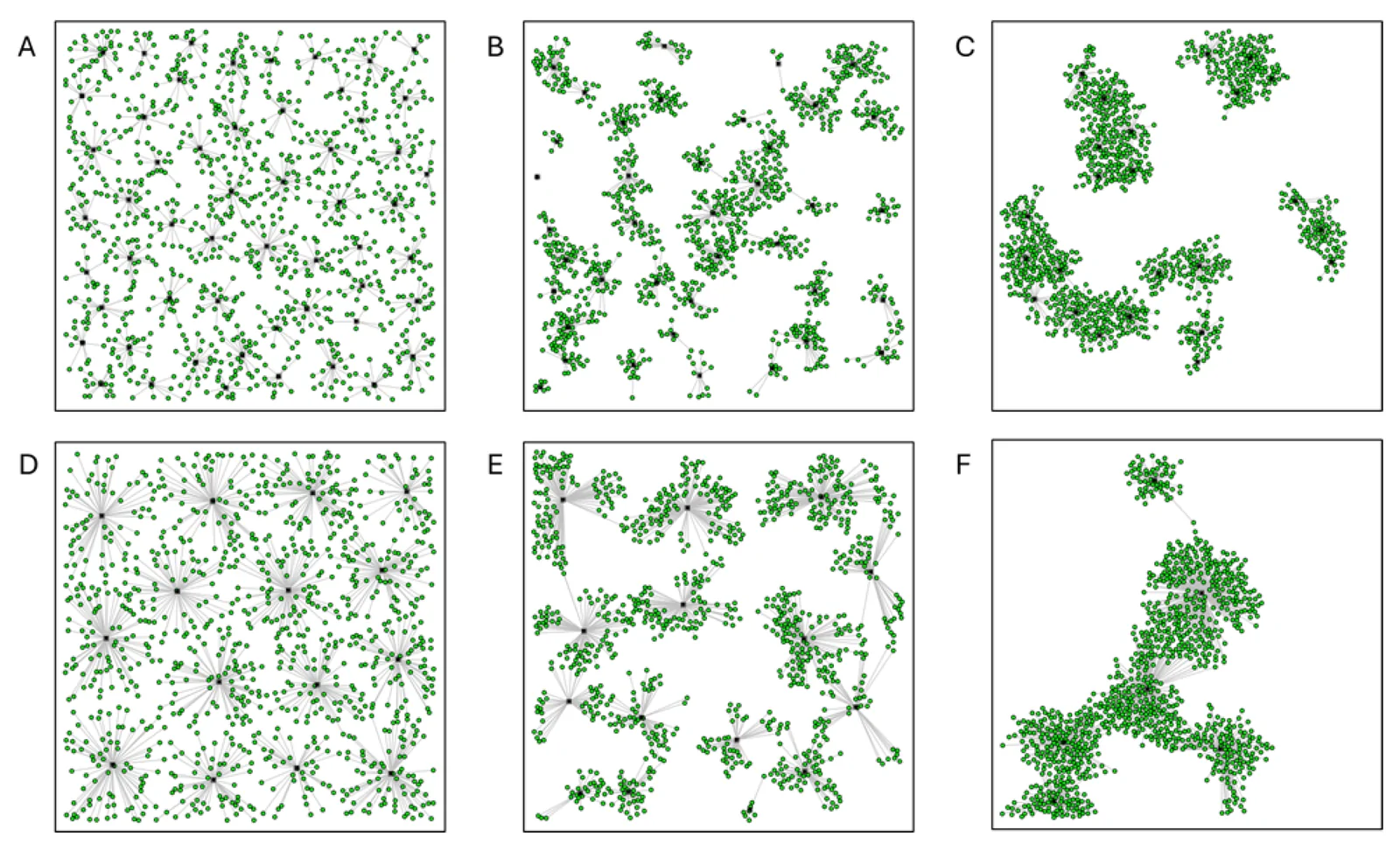
Examples to visually illustrate the differences in the resulting scenarios when different parameters are chosen. What is shown are the steady state solutions for varying cluster numbers ($N_{c}$; horizontally) and ranges ($R_{\max}$; top–bottom). The demand locations are shown as green disks, and the logistics robots are shown as black squares. The shown parameter values are as follows: $R_{\max}=100$ m (**A**–**C**) or $R_{\max}=200$ m (**D**–**F**). $N_{c}=1000$ (**A**,**D**), $N_{c}=100$ (**B**,**E**), or $N_{c}=10$ (**C**,**F**).

For the purposes of benchmarking, a first test was conducted in which neither the number of robots nor their service capacity $N_{\max}$ were limited. So, the parameters are the number of clusters $N_{c}$ (10, 100, or 1000) and the service radius $R_{\max}$ (100 m or 200 m). Figure 6 shows an illustration for each of the 6 combinations of these parameter values.

### 6.4. Performance Measures

Suitable performance indicators are the number of robots deployed when the system reaches its end state (all units are supplied), the evolution over time of the fraction of units being serviced, the distribution of units between logistics robots, and the distance between them, for the different combinations of parameter values. These can be used to evaluate the quality of the solution found by the distributed algorithm presiding over the self-deployment of the autonomous logistics force when faced with different constraints (distribution of units to be supplied).

### 6.5. Data Collection

The performance of the proposed approach has been studied using Monte Carlo simulation, the results of which are discussed in Section 7. The data collection involved $10^{5}$ runs per parameter combination. The reported values are the average values, the minimum and maximum values, and the standard deviation. With regard to the comparative analysis against using k-means, $10^{3}$ simulation runs were performed.

### 6.6. Simulation Setup

We conducted extensive Monte Carlo simulations within a controlled environment. Autonomous logistics robots are deployed within a predefined 1 ${\mathrm{km}}^{2}$ area, servicing distributed logistics platforms with fixed demands. The modeling does not account for the purpose or the nature of the platforms (civilian or military).

### 6.7. Bio-Inspired Algorithm Description

Our method, described in Section 5, adopts territorial dynamics similar to biological ecosystems. Each robot independently adjusts its position according to local demand, maintaining decentralized decision-making with minimal global input.

### 6.8. Experimental Setup

Simulations were executed by varying the number of clusters ($N_{c}$), the radius of service area ($R_{\max}$), and the number of field units to systematically assess algorithmic performance under diverse operational conditions.

### 6.9. Comparative Analysis

Because the method used is reminiscent of k-means clustering with a sequential element (adding one new logistics unit or cluster-head at a time) and a maximum range (in the standard k-means clustering method, a point is allocated to the nearest cluster, whatever the distance to the latter’s existing center), we conducted a partial comparative analysis (the results of which are discussed in Section 7.3).

## 7. Results

This Section discusses the results we obtained.

### 7.1. Impact of Demand Distribution

Our results demonstrate that the proposed bio-inspired territorial partitioning efficiently adapts to different demand distributions. We found that more aggregated distributions resulted in pronounced variability in individual robot workloads, suggesting a high degree of flexibility and robustness.

### 7.2. Performance Metrics

Quantitative analysis includes the number of robots required, service coverage over time, and load distribution among robots. The data indicates that bio-inspired territorial allocation can deliver performance on par with a traditional clustering method, such as k-means, and can do so at runtime and without preliminary offline calculations.

**Figure 7:**
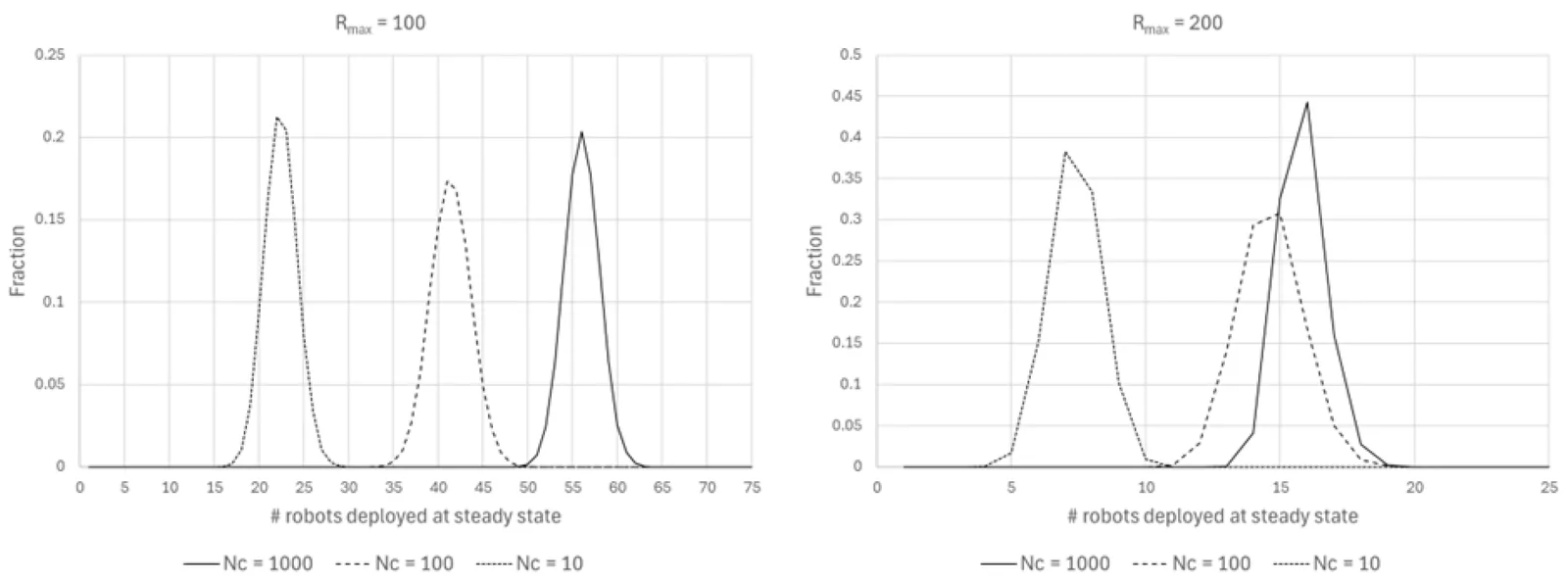
The distribution of the number of logistics robots deployed at steady state, for different spatial distribution patterns ($N_{c}=1000,100,10$) and two different maximum supply ranges ($R_{\max}=100$ m or 200 m). There are $10^{5}$ independent runs per combination of parameter values.

Figure 7 shows that the number of autonomous logistics robots that the algorithm estimates are necessary to supply all units varies substantially depending on their spatial distribution (homogeneous vs. aggregative). This is particularly the case when the maximum service range $R_{\max}$ is low (compare the left and right sub-figure in Figure 7).

**Figure 8:**
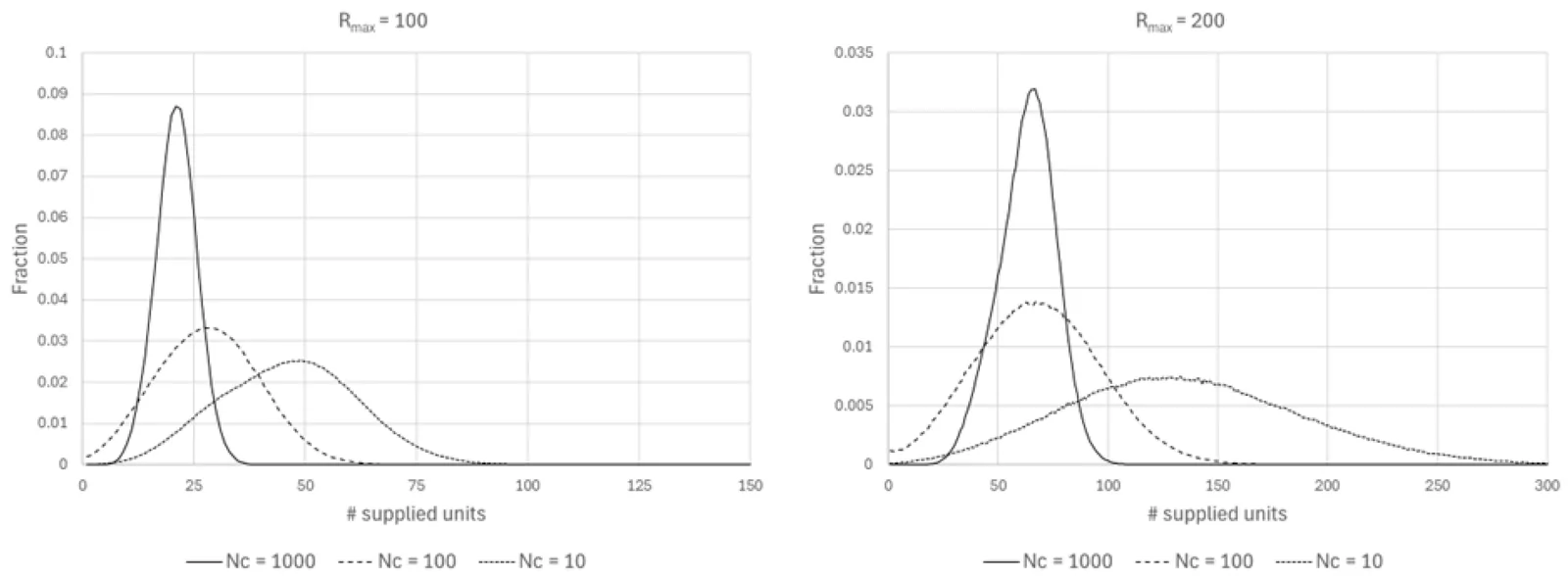
The distribution of the number of supplied units per logistics robot at steady state, for different spatial distribution patterns ($N_{c}=1000,100,10$) and two different maximum supply ranges ($R_{\max}=100$ m or 200 m). There are $10^{5}$ independent runs per combination of parameter values.

Figure 8 shows that, for a homogeneous distribution of units to be supplied ($N_{c}=1000$), the number serviced by each robot at steady state is quite stable (narrow peak). In scenarios where the demand distribution is increasingly aggregated, variability is much more pronounced, with that same number varying by up to an order of magnitude ($N_{c}=10$, $R_{\max}=200$ m). By comparing the left and right sub-figure in Figure 8, we see that the effect is clearly amplified by a wider catchment area (higher values of $R_{\max}$).

### 7.3. Comparative Analysis

The difficulty with our comparative analysis (cf. Section 6.9) is to determine what is a suitable ending/success criterion for the standard k-means clustering algorithm in the presence of the aforementioned maximum range limit and to identify a meaningful performance comparison variable. We opted for the following: *k* was set to the highest observed value obtained using our heuristic method (where the number of robots deployed, analogous to *k*, is a variable and not a parameter), as listed in Table 2. Note: the relative performance is likely to be improved for more advantageous values of k.

We then compared the distribution of cluster size (maximum and standard deviation; see Table 3), under the assumption that lower variability is beneficial, since it corresponds to a more homogeneous demand distribution.

**Figure 9:**
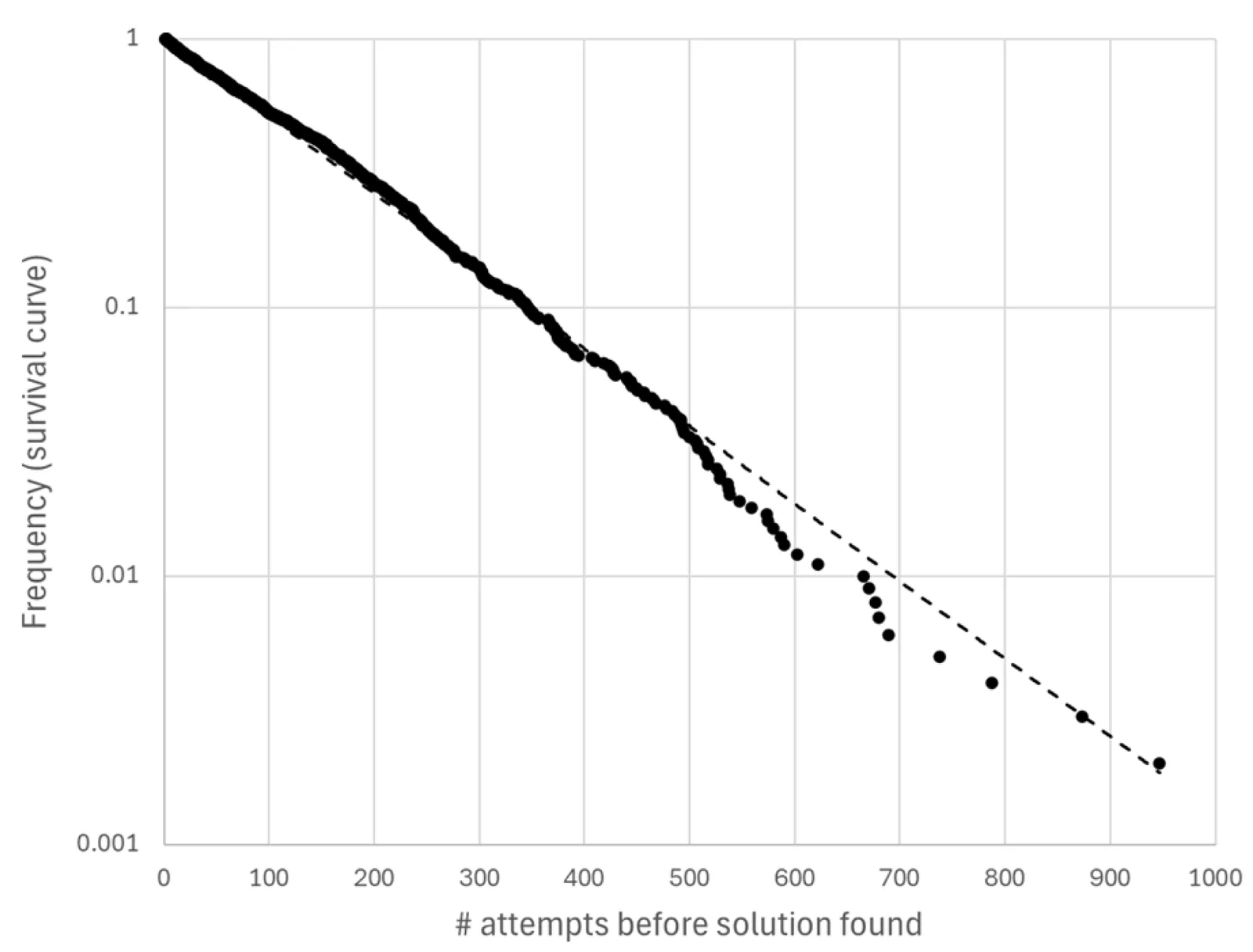
The survival curve showing an exponential decay signature in the number of attempts required to identify a valid solution (all units are supplied) using the modified k-means clustering method (k=65; see text for details). The half-life is ≈143 attempts. $R_{\max}=100$ and $N_{c}=1000$. The results shown are based on $10^{3}$ independently generated unit distributions.

Furthermore, since there is no guarantee that the *k*-means clustering standard method will result in a configuration in which every unit is supplied (considering the maximum range limit), we also performed a statistical analysis of the number of attempts required for such a solution to be discovered, for $R_{\max}=100$ and $N_{c}=1000$ (see Figure 9).

Overall, it was discovered that the k-means clustering standard method, when used with the conservative k= maximum number of logistics robots deployed for the same $R_{\max}$ and $N_{c}$ parameter values (cf. Table 2), tends to achieve a more homogeneous demand (repartition of units between robots) at the expense of substantially heavier computational load for high values of the $N_{c}$ parameter. For $R_{\max}=100$, only for highly clustered unit distributions ($N_{c}=10$) does the standard method return a superior solution at a comparatively low computational cost.

For $R_{\max}=200$, the standard method returns better results without incurring unsustainable computational loads across all $N_{c}$ values (see Table 4).

Besides being a centralized optimization method that requires complete information and would not be immediately implementable, it is worth noting that the performance comparison method strongly favors k-means clustering over distributed decision-making by selecting the highest value of *k* observed when using the latter.

For example, for $R_{\max}=100$ and $N_{c}=10$ (a combination of parameter values for which the standard method excels for k=30), it was found that an average of ≈72 attempts were needed to identify a solution when *k* was set to the average number of robots deployed by the heuristic method (k=22).

## 8. Conclusions and Reflections

This paper validates a bio-inspired approach for decentralized logistics, and the results presented suggest effectiveness of this approach across varying demand distributions (we acknowledge that for stronger claims, application-specific investigations should be undertaken which may include, e.g., more realistic models for signal attenuation, platform failure, etc.). Future work includes integration into realistic scenarios and exploration of hybrid human–autonomous logistics networks.

This article is as a contribution to a special issue focusing on *“Bio-Inspired Algorithms and Systems: From Nature’s Concepts to Scalable Solutions”*. For Technology Readiness Level (TRL) 9, operational aspects such as, e.g., system-wide communication or oversight will adversely impact scalability. It should therefore be remembered that the presented work is theoretical in nature (i.e., low-TRL) and is not making any claims with regard to high-TRL solutions to the challenge of (defense) logistics. Our work explores a novel approach to the problem, and our results show, within the simplified model of reality in which our simulation is conducted, that this approach has significant potential.

### 8.1. Contextualizing the Approach Within Broader Autonomous Logistics

Autonomous logistics represents an increasingly significant area within robotics and autonomous systems research, spanning applications from commercial and industrial domains to disaster response and healthcare. Traditional approaches in logistics generally involve centralized control, exhaustive global planning, and continuous monitoring from a central authority. Centralization enables comprehensive optimization based on a complete overview but introduces vulnerabilities including limited scalability, susceptibility to bottlenecks, latency in decision-making, and heavy dependence on reliable and continuous communication networks.

In contrast, the decentralized autonomous logistics approach outlined here reduces dependence on global communication and centralized control, emphasizing localized decision-making and adaptation. This decentralization emerges as highly desirable in environments where continuous or reliable global communication is difficult, expensive, unreliable, or deliberately disrupted.

For instance, in disaster response scenarios, centralized communication infrastructure often fails or becomes overloaded due to increased demand, rendering traditional logistical coordination ineffective. Similarly, in defense or contested environments, communication signals may be deliberately jammed or disrupted, making reliance on centralized control vulnerable and impractical. In this context, logistics strategies that utilize decentralized decision-making, such as those inspired by territorial behavior observed in nature, are advantageous because they inherently limit communication needs, relying predominantly on local interactions and self-organization. Each autonomous robot, operating independently, reduces the risk of communication infrastructure overload, thereby maintaining system integrity even in degraded environments. Decentralized solutions also demonstrate higher adaptability and resilience, as local decisions can quickly adapt to rapidly changing circumstances without requiring global recalculations or transmissions.

### 8.2. The Connection Between Biological Inspiration and the Field of Logistics

The bio-inspired territorial allocation method described here aligns with such decentralized strategies. It draws inspiration from natural phenomena like territorial partitioning by animals and insects, which autonomously organize and adapt their spatial distributions based on local conditions without centralized coordination. Just as competing predators naturally adjust their territorial boundaries based on resource density and individual strength, autonomous logistics robots adjust territories based on the intensity of local demand and their remaining resource capacities. This results in a flexible and adaptive system capable of efficiently managing resources under dynamically shifting and uncertain conditions.

### 8.3. Preliminary Insights into the Scalability of the Approach

In the presented algorithmic framework, the departure of supply units is sequential, i.e., another robot only departs the central depot (if there are still some unsupplied field units) when its predecessor has reported having reached its locally optimal destination. This is currently the main limitation to scalability (as the time to reach steady state grows linearly with the number of robots required to achieve full coverage, which is itself a function of the number and deployment area of field units). Preliminary tests were conducted for a synchronized departure (i.e., a fixed number of robots leave the depot simultaneously), but the results are not presented here, primarily because we wanted to keep the number of supply units deployed as a dynamic variable (as opposed to a fixed parameter) to study the influence of field units’ distribution.

### 8.4. Reflecting on the Simplified Topology and Landscape

We acknowledge the simplified environment used in our investigation. To amend our model to a more realistic landscape, simple spatial reasoning techniques (e.g., shortest path to the intended destination node in a patchy overlay network) could be applied to navigate an environment with known obstructions. In the case of unknown environments, newly discovered obstacles would need to be dealt with autonomously. Signal attenuation would of course need to be considered in any real deployment scenario. In some cases, it could actually help simplify the optimization problem, at the risk of some field units that were not able to communicate their needs and position remaining unsupplied. Such units being discovered by the supply robots as they move closer is in fact partially and indirectly considered in the manuscript, since movement decisions (directional changes) are based on which field units are within supply range of the robot at any given time. This in fact implies that supply and communication ranges are the same and identical for all field units (since the robot ignores those beyond that range), but this could easily be relaxed in future work to model interaction more realistically. However, this appears out of scope for the present proof-of-concept paper.

### 8.5. Considerations on Potential Future Work

A number of open issues remain, and before this work can be taken to a higher TRL, these should be addressed or investigated. Among these issues are the following:

As stated in Section 2, the study of $N_{\max}$, the parameter controlling the maximum number of clients a platform can provide service to, will be the subject of future work.

As discussed in Section 2.1, we only consider a single base, but extending the work to multiple bases as well as to using finite resources at the bases is an interesting extension to our work. As stated, we do not expect the outcomes to be significantly different in the general case, so any work directed at this should also come with a well-defined specific use-case where there are additional dimensions to the problem which can be addressed by an amended approach/which may impact the performance of our approach.

Furthermore, as discussed above (Section 8.3) in the context of the scalability of the approach, synchronous deployment was considered a separate investigation topic to be the subject of future work. As mentioned, this should probably come as part of investigations considering a well-defined and specific use-case.

Finally, as mentioned in Section 8.4, we have used a simplified model for the environment, as operating with unknown obstacles would require a certain degree of autonomy from the individual robot. This is, however, a completely separate research question that is out of scope for the present article. Future work could focus first on how the problem definition and complexity changes when we assume operations in an unknown environment (considering aspects such as, e.g., signal attenuation, noise, or intentional interference) and then explore approaches for autonomous robots.

### 8.6. Conclusions

The overarching goal addressed by bio-inspired logistics research is offering highly adaptive, robust, and efficient logistical solutions, capable of coping with the inherent unpredictability and complexity of real-world environments. The central challenge is thus to develop decentralized logistical systems capable of dynamically organizing themselves to efficiently meet evolving demands.

This can be achieved through innovative algorithms and methods inspired by biological systems, due to their inherent robustness, adaptability, and decentralized decision-making capabilities. This article shows that a bio-inspired approach for decentralized logistics can offer effectiveness across varying demand distributions and as such lends itself as a basis for solutions tailored to specific logistic challenges and problems.

## Figures and Tables

**Table 1:** A comparison of the 4 use-cases on some key aspects.

Use-Case	Provides	Landscape	Deployment	Operational Limitations	QoS Measure (per Customer)
Wi-Fi Access	Service	Static	Finite, Limited	Resources required to operate the robot and the service	Distance to server (robot) Connectivity of Robot
Pre-emptive supply distribution	Physical goods	Dynamic	Finite, Limited	The available stock of resources carried by the robot	Suitability of stock provided
Continuous operation	Either	Either	Indefinite	Either	Ability to re-supply over time
Combat logistics	Either	Either	Indefinite	Either	Ability to re-supply over time

**Table 2:** Key performance statistics for **supply** (cf. Table 3 for the demand). The number of logistics robots deployed, calculated from $10^{5}$ independent runs per parameter value combination are shown.

			Mean	Minimum	Maximum	St. Dev
	$N_{c}=$	1000	56.02	48	65	1.97
$R_{\max}=100$	$N_{c}=$	100	41.39	32	51	2.27
	$N_{c}=$	10	22.43	15	30	1.84
	$N_{c}=$	1000	15.81	13	21	0.87
$R_{\max}=200$	$N_{c}=$	100	14.63	10	20	1.22
	$N_{c}=$	10	7.38	4	11	0.96

**Table 3:** Key performance statistics for **demand** (cf. Table 2 for the supply). Shown are the number of clients supplied by each robot, over $10^{5}$ independent runs per parameter value combination.

			Mean	Minimum	Maximum	St. Dev
	$N_{c}=$	1000	17.85	1	46	5.52
$R_{\max}=100$	$N_{c}=$	100	24.16	1	84	11.76
	$N_{c}=$	10	44.58	1	120	15.77
	$N_{c}=$	1000	63.24	6	126	29.54
$R_{\max}=200$	$N_{c}=$	100	68.36	1	215	44.69
	$N_{c}=$	10	135.52	1	396	91.05

**Table 4:** Key performance variables statistics for k-means clustering for comparison with Table 3. What is shown are the number of clients supplied by each individual robot, calculated from $10^{3}$ independent runs per parameter value combination.

			Minimum	Maximum
	$N_{c}=$	1000	1	39
$R_{\max}=100$	$N_{c}=$	100	1	69
	$N_{c}=$	10	3	99
	$N_{c}=$	1000	14	95
$R_{\max}=200$	$N_{c}=$	100	1	157
	$N_{c}=$	10	8	278

## Data Availability

No new data were created or analyzed in this study. Data sharing is not applicable to this article.
